# Editorial: Advancements in prenatal diagnosis: from noninvasive prenatal tests to novel fetal imaging

**DOI:** 10.3389/fmed.2025.1682161

**Published:** 2025-09-29

**Authors:** Sarah M. Cohen, Min Chen, Liqun Sun

**Affiliations:** ^1^Department of Obstetrics and Gynecology, Hadassah – Hebrew University Medical Center, Jerusalem, Israel; ^2^Department of Obstetrics and Gynecology, The Third Affiliated Hospital of Guangzhou Medical University, Guangzhou, Guangdong, China; ^3^Key Laboratory for Major Obstetric Diseases of Guangdong Province, Department of Fetal Medicine and Prenatal Diagnosis, The Third Affiliated Hospital of Guangzhou Medical University, Guangzhou, Guangdong, China; ^4^Institute of Medical Genetics and Development, Key Laboratory of Reproductive Genetics (Ministry of Education) and Women's Hospital, Zhejiang University School of Medicine, Hangzhou, China; ^5^Department of Ultrasound, Women's Hospital, Zhejiang University School of Medicine, Hangzhou, China

**Keywords:** prenatal diagnosis, NIPT, Niemann-Pick disease, chromosomal micro array, chorionic villus sampling, double aortic arch, fetal growth restriction (FGR), hypoxemia

## Introduction

This Research Topic presents a curated collection of seven articles highlighting recent advances in prenatal diagnosis, fetal medicine, and perinatal outcomes. The articles span a spectrum of innovations and challenges in obstetric imaging, non-invasive testing, immunological mechanisms, and genetic risk assessment. Together, these studies reflect the growing precision and complexity of prenatal and perinatal care, encompassing both technological progress and the nuanced clinical decisions that arise in high-risk pregnancies. The summaries that follow showcase each study's core contributions and are interwoven to illustrate the broader themes of diagnostic accuracy, clinical relevance, and translational potential.

The first five articles in this Research Topic address key advancements in diagnosis and prognostication in fetal medicine. These include innovations in genetic screening, ultrasonographic assessment, and comparative procedural outcomes, all of which are aimed at improving the accuracy and clinical relevance of prenatal care.

To begin, a succinct mini review conducted by Eltabbakh et al. examined the evolving role of obstetricians in the context of non-invasive prenatal testing (NIPT), which has expanded beyond trisomy screening to include microdeletions and single-gene disorders. The authors highlighted the psychosocial and ethical dilemmas associated with routine NIPT uptake, especially in low-risk pregnancies, and emphasized the importance of genetic counseling to mitigate anxiety and ensure informed decision-making. A key technical focus is the significance of fetal fraction in cfDNA testing and its implications for test accuracy. Obstetricians are positioned as crucial mediators between complex genetic technologies and personalized patient care.

A proof-of-concept case series conducted by Lau et al. introduced a non-invasive approach to screen for autosomal recessive Niemann-Pick disease type C1 using cell-free fetal DNA (cffDNA). Leveraging amplicon-based NGS, the assay accurately identified NPC1 status in three high-risk pregnancies with known familial variants. The results were consistent with those of invasive testing, highlighting the feasibility of early, non-invasive detection of AR disorders. The authors advocated for the broader implementation of targeted cffDNA screening in genetically at-risk populations, emphasizing its potential to reduce parental anxiety and inform decision-making earlier during gestation.

A large-scale retrospective study conducted by Huang et al. analyzed 2,272 singleton pregnancies in women under 35 to evaluate the association between varying nuchal translucency (NT) measurements and chromosomal abnormalities. Using CMA and CNV-seq, the authors showed that NT as low as 2.5 mm is associated with an increased risk—especially when combined with soft markers or structural anomalies. The study provides critical data that supports genetic testing thresholds and reinforces the need for nuanced risk assessment, even among younger maternal populations that are traditionally viewed as low risk.

A retrospective cohort study conducted by Kim et al. compared 200 chorionic villus sampling (CVS) cases and 498 amniocentesis cases in terms of pregnancy and child outcomes. Although both groups showed similar obstetric and neurodevelopmental outcomes, CVS was associated with a higher risk of cervical cerclage due to a short cervix. The findings suggest that while both procedures are generally safe, CVS may warrant closer cervical monitoring. These results emphasize the importance of personalized counseling and selecting the most appropriate invasive test based on individual patient factors.

A separate retrospective study conducted by Jiang et al. evaluated 31 fetuses with double aortic arch (DAA) and proposed quantitative ultrasound markers—including tracheal diameter Z-scores and arch pinch angles—as predictors of postnatal symptomatology. The data demonstrate that smaller tracheal diameters and tighter angles correlate with symptomatic presentations, and thresholds are proposed to guide prenatal counseling and surgical planning. This study supports the role of detailed fetal echocardiography not only for diagnosis but also for predicting clinical outcomes and stratifying risk.

In addition to the clinically focused studies, two articles in this Research Topic used basic science—one with a sheep model and the other with *in vitro* systems—to deepen our understanding of key biological mechanisms. These insights hold promise for refining future clinical interventions.

In an experimental sheep model of fetal growth restriction, Darby et al. utilized MRI-based phase-contrast and T2 oximetry to assess how chronically hypoxemic fetuses respond to an additional acute hypoxic event. The authors showed that while chronic hypoxia leads to adaptive preferential streaming of oxygenated blood to the brain, this mechanism is insufficient to protect cerebral oxygen delivery during acute stress. The study emphasizes the complex and layered vulnerability of the FGR fetus and the potential long-term consequences for hepatic and pulmonary function postnatally.

Ames et al. developed a novel whole blood platelet phagocytosis assay (WHOPPA) that simulates anti-HPA-1a-mediated fetal platelet destruction, the hallmark of fetal and neonatal alloimmune thrombocytopenia (FNAIT). The assay revealed that specific monocyte subsets are primarily responsible for phagocytosis and that effector-silenced monoclonal antibodies can inhibit this process. This tool could become instrumental in risk stratification and therapeutic screening, offering a translational bridge between bench research and the clinical management of FNAIT.

## Conclusion

Taken together these seven studies underscore a paradigm shift in fetal medicine, moving from protocol-driven algorithms to more nuanced, evidence-based decision-making that integrates molecular diagnostics, biophysical imaging, and individualized risk profiling. Whether refining diagnostic thresholds for NT, modeling fetal oxygenation in FGR, implementing novel functional assays in alloimmunity, or expanding the scope of NIPT to include its application to single-gene and autosomal recessive conditions, these contributions advance the goal of earlier, safer, and more precise prenatal care. The collective findings offer valuable guidance for clinicians, researchers, and policymakers who aim to optimize perinatal outcomes across diverse clinical scenarios.

The global implications of these innovations are profound: the push toward precision medicine must be adapted to settings with limited resources to ensure that advances in screening, prognostication, and risk stratification are accessible and ethically implemented across diverse populations. Ultimately, these studies highlight the necessity of integrating technological progress with ethical vigilance and psychosocial support—a combination that is essential for optimizing perinatal outcomes and addressing disparities in prenatal care worldwide.

